# Myogenic Precursors from iPS Cells for Skeletal Muscle Cell Replacement Therapy

**DOI:** 10.3390/jcm4020243

**Published:** 2015-01-29

**Authors:** Isart Roca, Jordi Requena, Michael J. Edel, Ana Belén Alvarez-Palomo

**Affiliations:** 1 Control of Pluripotency Laboratory, Department of Physiological Sciences I, Faculty of Medicine, University of Barcelona, Hospital Clinic, Casanova 143, 08036, Barcelona, Spain; E-Mails: isart.roca@gmail.com (I.R.); joreos@hotmail.com (J.R.); michaeledel@ub.edu (M.J.E.); 2 Faculty of Medicine, University of Sydney Medical School, Division of Pediatrics and Child Health, Westmead Children’s Hospital, Locked Bag 4001, Westmead NSW 2145, Sydney, Australia; 3 School of Anatomy Physiology & Human Biology and The Harry Perkins Institute for Medical Research (CCTRM), the University of Western Australia, 6 Verdun St, Nedlands WA 6009, Perth, Australia

**Keywords:** induced pluripotent stem cells, skeletal muscle, muscular dystrophy, myogenic progenitors, stem cell therapy

## Abstract

The use of adult myogenic stem cells as a cell therapy for skeletal muscle regeneration has been attempted for decades, with only moderate success. Myogenic progenitors (MP) made from induced pluripotent stem cells (iPSCs) are promising candidates for stem cell therapy to regenerate skeletal muscle since they allow allogenic transplantation, can be produced in large quantities, and, as compared to adult myoblasts, present more embryonic-like features and more proliferative capacity *in vitro*, which indicates a potential for more self-renewal and regenerative capacity *in vivo*. Different approaches have been described to make myogenic progenitors either by gene overexpression or by directed differentiation through culture conditions, and several myopathies have already been modeled using iPSC-MP. However, even though results in animal models have shown improvement from previous work with isolated adult myoblasts, major challenges regarding host response have to be addressed and clinically relevant transplantation protocols are lacking. Despite these challenges we are closer than we think to bringing iPSC-MP towards clinical use for treating human muscle disease and sporting injuries.

## 1. Introduction

Skeletal muscle is a dynamic organ in which an efficient regeneration process ensures repair after damage. The process of muscle regeneration creates new myofibers after necrosis resulting from injury or a degenerative process. The myonuclei of multinucleated myofibers are post mitotic, arrested in the G_0_ phase of the cell cycle and unable to proliferate. A resident population of adult myogenic stem cells called “satellite cells” is the main player in the regeneration process. These cells reside in a quiescent state, located between the basal membrane and the plasmalemma of each myofiber. Upon signaling from the damaged myofibers, satellite cells become activated, undergo an asymmetric division to self-renew, and produce activated myoblasts that are able to proliferate, migrate to the site of injury, and fuse with the existing myofibers or to form new myotubes [1]. Besides satellite cells, other populations with stem cell properties have been described as capable of undergoing myogenesis and contribute to myofiber repair, such as mesangioblasts, bone marrow-derived stem cells, pericytes, or interstitial muscle-derived stem cells, though it appears that *in vivo* they contribute to a much smaller extent than satellite cells [2]. 

Repeated cycles of myofiber necrosis and regeneration in muscle dystrophies (MD), such as Duchenne muscular dystrophy (DMD) and some limb girdle dystrophies, result in exhaustion of satellite cell regenerative capacity in humans [3]. Similarly, neuromuscular diseases in which neuromuscular junctions are lost and muscles undergo subsequent atrophy, such as spinal muscle atrophy (SMA) and familiar amyotrophic lateral sclerosis (ALS), present deficiencies in the satellite cells compartment [4,5]. Moreover, the myofibers in both MDs and neuromuscular diseases present different abnormalities in their structure and functionality [6,7,8]. Other situations in which muscle regeneration is compromised are severe injury [9] and inflammatory myopathies [3]. Restoration of the satellite cell compartment with healthy cells would restore the regenerative capacity of the muscle and progressively substitute the defective myofibers. Therefore, in all of these conditions, myogenic cell replacement therapy provides a promising perspective for the treatment of degenerative myopathies.

## 2. Using Myoblasts as a Cell Therapy

Transplantation of donor myoblast or satellite cells isolated from healthy individuals has been tried extensively in the past with somewhat positive but insufficient results and scarce references to functional improvement [10]. In 1995, allogenic normal myoblasts were transferred into the biceps brachii arm muscles of DMD patients in order to restore the lack of dystrophin protein [11]. Although some fusion of donor nuclei into host myofibers was observed, there was no significant improvement in muscle function. Genetic correction has also been explored to allow for autologous transplantation of expanded myoblasts, but results again showed engraftment but a low contribution to host fibers [12]. Massive death of most of the transplanted cells within a few days after intramuscular delivery has been reported by several laboratories [13]. The reasons why the myoblasts die initially are not clear but probably relate to immune aspects, anoikis, and a hostile environment in the host damaged muscle. Moreover, using myoblasts as a donor source poses a limitation in the amount of original tissue for cell isolation from normal human muscle biopsies. It also limits the possibilities of *in vitro* expansion because myoblasts are limited to a few passages due to senescence and the decreased self-renewal capacity of the cells due to the expansion process [14]. Therefore, it is difficult to obtain a clinically relevant number of transplantable myoblasts from a donor source. The use of other adult stem cells, with high proliferative capacity, as an alternative source of myogenic cells has been investigated with disappointing or inconclusive results such as bone marrow-derived stem cells [15], pericytes [16], and mesangioblasts [17]. Further research is needed to establish the efficacy of cell therapy using these types of donor cells.

Clinical trials using myogenic cell therapy to treat muscular dystrophies started in the 1990s, showed some engraftment of the donor cells but no clear signals of disease recovery or symptom alleviation (see Table 1). 

However, extensive preclinical and clinical work over the past few decades has helped to identify some relevant issues to address in order to improve cell therapy in muscular dystrophies. The main limitations of this therapy are transplanted cell engraftment and contribution to host myofibers, which seems to be highly dependent on survival—immunosuppression is thus required but other factors might be contributing as well—and migration out of the site of injection. The transplantation regime can also affect engraftment success [18]. 

Taking all this into account, the ideal donor cell for skeletal muscle regeneration should be easily accessible and able to expand extensively without losing myogenic and engraftment capacity, have a great survival and fusion rate with host myofibers (high myogenic capacity), and be highly motile to spread within the muscle. Moreover, it should contribute to the satellite cell compartment, enabling indefinite muscle regenerative capacity. Finally, the ideal myogenic donor cell should have low immunogenicity, and be able to be delivered systemically, since intramuscular injection does not seem a feasible approach given the large volume of muscle tissue to be treated.

However, extensive preclinical and clinical work over the past few decades has helped to identify some relevant issues to address in order to improve cell therapy in muscular dystrophies. The main limitations of this therapy are transplanted cell engraftment and contribution to host myofibers, which seems to be highly dependent on survival—immunosuppression is thus required but other factors might be contributing as well—and migration out of the site of injection. The transplantation regime can also affect engraftment success [18]. 

Taking all this into account, the ideal donor cell for skeletal muscle regeneration should be easily accessible and able to expand extensively without losing myogenic and engraftment capacity, have a great survival and fusion rate with host myofibers (high myogenic capacity), and be highly motile to spread within the muscle. Moreover, it should contribute to the satellite cell compartment, enabling indefinite muscle regenerative capacity. Finally, the ideal myogenic donor cell should have low immunogenicity, and be able to be delivered systemically, since intramuscular injection does not seem a feasible approach given the large volume of muscle tissue to be treated.

**Table 1:** Clinical trials using myogenic progenitors for the treatment of Duchenne’s muscular dystrophy.

Year	*N*	Donor Cells	Injection	Immuno-Suppression	Results	Conclusions	Reference
1992	4	Allogeneic immunocompatible myoblasts	Intramuscular: tibialis anterior, biceps brachii, and/or extensor carpi radialis longus	No	Variable response. Hybrid myofibers and modest strength increase in 3 of the 4 patient. Slow decay over time.	No signs of immune rejection	[19]
1992	8	Allogeneic immunocompatible myoblasts	Intramuscular: tibialis anterior	Cyclosporin	PCR evidence of hybrid fibers after 1 moth for 3 patients (1 patient tested still positive after 6 months).	Younger patients with less fibrosis presented best outcomes	[20]
1993	5	Allogenic myoblasts	Intramuscular: biceps brachii, left tibialis anterior	No	0%–36% hybrid fibers after 1 month. Low dystrophin expression. Strong decrease in hybrid fibers at 6 months. No functional recovery.	Transplantation cannot be done without immuno-suppression	[21]
1993	8	Allogeneic myoblasts	Intramuscular: biceps brachii	Cyclosporin	Poor functional recovery and lack of donor-derived dystrophin.	Younger donor cells, regeneration induction and basal laminal fenestration could improve results	[22]
1993	1	Asymptomatic twin sibling myoblasts	Intramuscular: extensor carpi radialis, biceps	No	After 1 year, significant force gain (12%–31%) in wrist extension but not for elbow flexion. Small increase in dystrophin positive and type II fibers.	Small benefit may be due to a low level of spontaneous muscle regeneration	[23]
1995	12	Allogeneic myoblasts	Intramuscular: biceps brachii Injection repeated monthly over 6 months	With and without Cyclosporin	There was no significant change in muscle strength. % of hybrid fiber varied between 10.3 (1 patient), 1 (3) and 0 (8).	Patient age did not correlate with outcome	[11]
1997	10	Allogeneic immune-compatible myoblasts	Intramuscular: tibialis anterior	Cyclosporin	Myoblast survival after 1 month in 3 patients and after 6 month in 1 patient. No recovery symptoms or clinically significant dystrophin expression.	-	[24]
2004	3	Allogeneic myoblasts	Intramuscular: tibialis anterior	Tacrolimus	Hybrid fibers observed in all 3 patients (9%, 6%, 8% and 11%)	-	[25]
2006	9	Allogeneic immuno-compatible myoblasts	Intramuscular Tibalis anterior. High density injections	Tacrolimus	At 4 weeks, 3.5%–26% hybrid fibers	Dystrophin expression restricted to injection site and mostly in short inter-injection distances	[26]
2007	1	Allogeneic myoblasts	Intramuscular Thenar eminence, biceps brachii and gastrocnemius High density injections	Tacrolimus	At 18 months, 34.5% hybrid myofibers in gastrocnemius but almost 0% in biceps brachii. Increased strength only observed in thumb.	-	[27]
On-going	-	Mesoan-gioblasts	Intra-arterial	Tacrolimus	Not yet	-	*

* EudraCT Number: 2011-000176-33; Sponsor Protocol Number: DMD03; Start Date *: 14 February 2011; Sponsor Name: FONDAZIONE CENTRO S; RAFFAELE DEL MONTE TABOR; Full Title: Cell Therapy of Duchenne Muscular Dystrophy by intra-arterial delivery of HLA-identical allogeneic mesoangioblasts.

## 3. Induced Pluripotent Stem Cells (iPSCs)-Derived Myogenic Progenitors (iPSC-MP)

Embryonic stem cells (ESC) are pluripotent stem cells derived from the inner cell mass of a blastocyst that are able to self-renew and to be differentiated in all tissues in the body. Induced PSCs share most of the features of ESCs but are derived from adult somatic cells, e.g., dermal fibroblasts, by the transient expression of a defined set of reprogramming factors [28]. The fact that iPSCs do not involve the destruction of embryos, with the consequent ethical issues, and allow for autologous production of the pluripotent cells has opened up an enormous range of possibilities for the regenerative cell therapy field. Since iPSCs have limitless replicative capacity *in vitro* and can differentiate into myoblast-like cells, they represent an attractive source of myogenic donors for muscle regeneration. Induced PSC-MP also represents a highly valuable tool for *in vitro* drug testing and disease modeling for muscular genetic conditions that were so far limited because of the difficulties of obtaining large quantities of tissue.

Initially, human ESCs (hESCs) proved to be difficult to differentiate into myogenic progenitors, probably due to the fact that paraxial mesoderm and subsequently the myogenic program are not well recapitulated during embryoid body (EB)—three-dimensional aggregates of pluripotent stem cells—formation [29]. The first protocols using different sequential culture conditions, including a mesenchymal differentiation step, were successful at producing myogenic progenitors capable of engrafting *in vivo* but these protocols were lengthy and inefficient [30]. It has been reported that the need for a mesodermal transition previous to a myogenic commitment is determined by the epigenetic landscape in human ESCs [31]. Higher efficiency and shorter protocols were designed by overexpression of myogenic transcription factors. Pax3 and Pax7 are paired box transcription factors that contribute to early striated muscle development and are expressed in the dermatomyotome of paraxial mesoderm. Darabi and colleagues showed that inducible expression of Pax3 using viral vectors at early EB formation overcame mesoderm patterning restrictions and yielded up to 50% myogenic cells within barely a week [29]. Albini *et al.* described how overexpression of MyoD1—a transcription factor that appears after Pax3 and Pax7 in muscle development and in activated satellite cells—alone could not induce myogenic commitment directly on hESCs, but concomitant overexpression of the chromatin remodeling complex component BAF60C overcame the mesodermal transition limitation [32]. In opposition to these results, Rao *et al.* describe hESC-derived myogenic progenitors by inducible lentiviral overexpression of MyoD1 directly on hESC cells, without a previous EB formation [33].

Other more efficient and genetic modification-free protocols have been described to obtain myogenic progenitors from hESCs, such as isolation of the PDGFRα^+^ population from EB derived-paraxial mesoderm [34] or isolation of the SM/C-2.6^+^—satellite cell-like—population from differentiating mouse ESC-derived EB cultured in high serum [35]. 

Since the appearance of iPSCs, extensive work has been done to obtain myogenic progenitors with a vision to their clinical application and disease modeling (Table 2). The first iPSC-MP came from mouse cells using a protocol similar to the one described above for ESC [35], based on spontaneous differentiation and sorting of SM/C2.6 positive cells [36]. Similarly, the group of Awaya reported a method of deriving mesenchymal cells with myogenic capacity from EB by a protocol based on selective enrichment though step-wise culture conditions [37]. The resulting cells showed long-term engraftment in immunocompromised mice pre-injured with cardiotoxin, and evidence of replenishing the satellite cell compartment. However, these protocols are long and not very efficient. Using an inducible lentiviral expression system, Darabi *et al.* produced satellite cell-like progenitors by overexpression of Pax7—a transcription factor required for somite myogenesis in the embryo and a marker for satellite cells in the adult—in EB from mice (miPSCs) and humans (hiPSCs) [38,39]. The resulting cells were able to engraft in a mouse model of muscular dystrophy and to produce regeneration and restore some muscle strength, and even showed evidence of donor-derived satellite cells—by expression of Pax7 and M-cadherin by the capacity of regeneration after a subsequent injury. They reported much better proliferative capacity of the myogenic progenitors *in vitro* and much better engraftment as compared to myoblasts. Lentiviral inducible overexpression of Pax3 in iPSCs from dystrophin-lacking mice, which were gene corrected with a truncated version of dystrophin (μ-utrophin), produced in a similar fashion myogenic progenitors that engrafted, differentiated, and repopulated the satellite cell compartment and exhibited neuromuscular synapses [40]. Goudenege and colleagues described a two-step protocol consisting of first culturing in a myogenic medium and then infecting with an adenovirus expressing MyoD1 that rendered myogenic progenitors able to engraft in the muscular dystrophy model mdx mice [41]. Also, using a self-contained, drug-inducible expression vector, based on the PiggyBac transposon for overexpression of MyoD1 and an efficient and quick conversion of undifferentiated iPSCs into myogenic progenitors with the ability to engraft in immunocompromised mice has been described [42]. A limitation on the use of MyoD1 for generating myogenic progenitors is the induction of cell cycle arrest when expressed too long at high levels; therefore, as an excellent proliferative capacity is needed to expand *in vitro* and survive *in vivo*, careful dosage and timing are necessary when using this transcription factor. 

Though gene overexpression approaches are fast, efficient, and appropriate to generate myogenic precursors for disease modeling, the risk of undesired genetic recombination or reactivation makes them unsuitable for a future application in the clinic for regenerative cell therapy. Different ways to obtain transplantable myogenic progenitors that do not involve any genetic modification and are still efficient and fast have recently been described. Recently, several reports describe other protocols without gene overexpression that include high concentrations of bFGF and EGF on free floating spheres [32] and, faster and more efficient, the use of GSK3 inhibitors and bFGF [43,44] in one of the cases, producing myogenic progenitors that engrafted in immunocompromised mice that contributed to the satellite cell pool [43]. 

Another way of avoiding introducing exogenous DNA is the transfection of *in vitro*-synthesized mRNA to overexpress the required transcription factors for myogenic conversion. It was recently shown as a proof of principle that transfection of MyoD1 mRNA in hiPSCs produced myogenic cells with the ability to fully differentiate [45] *in vitro*.

Other cells with myogenic potential that are not myoblasts have been derived from iPSCs: the group of Tedesco has developed mesangioblast (pericyte progenitors)-like cells that have been tested in animal models [46].

**Table 2:** Protocols for myogenic progenitor derivation from iPSC and *in vivo* testing.

Origin	Method	Myogenic Cells	Mice	Fiber Contribution	Satellite Cell	Ref.
miPSC	EB on high serum, culture on Matrigel+ SM/C2.6 Ab^+^ selection	Myoblast-like SM/C2.6^+^	- Irradiated mdx mice - Intramuscular - Cardiotoxin	- 58% fibers positive	Yes	[36]
hiPSC	EB + general differentiation +MyoD1 mRNA	Myoblast-like MyoD1^+^	No	-	-	[45]
miPSC	Inducible Pax7 expression on EB+ PDGFαR^+^FLK1^−^ selection	Myoblast-like PDGFaR^+^FLK1^−^	- Immuno-deficient - Intramuscular - Cardiotoxin	- 15%–20% fibers positive - Functional improvement	NA *	[38]
LGMD2D hiPSC	Inducible lentiviral MyoD1 on iPSC-derived MAB-like	MyoD1 expressing mesangioblast- like	- Immuno-deficient - Intramuscular (1) - Intra-arterial (2)	- (1) 53% fibers positive - (2) Muscle colonization	NA	[46]
hiPSC	EB+ITS medium + myogenic medium	Myoblast-like MyoD1^+^, Pax7^+^, Myf 5^+^	- Irradiated immuno-deficient - Intramuscular - Cardiotoxin	- 10%–17% fibers positive	Yes	[37]
hiPSC	Inducible Pax7 expression on EB	Pax7^+^ myoblast-like	- Immuno-deficient control (1) - immuno-deficient mdx (2) - Intramuscular - Cardiotoxin (1)	(1) Yes (2) Yes (2) Functional improvement	Yes	[39]
DMD **-hiPSC	Mesenchyal-like lineage differentiation +adenoviral MyoD1 expression	Myoblast-like MyoD1^+^	- Mdx mice - Intramuscular - Cardiotoxin	Yes	NA	[41]
hiPSC	EB on Matrigel, GSK3 inh., forskolin, bFGF STEMdiff APEL medium	Myoblast-like MyoD1^+^, Pax7^+^, Myf 5^+^, Gata2^+^	- Immuno-deficient - Intramuscular - Cardiotoxin	Yes	Yes	[44]
hiPSC	ITS Medium+ GSK3 inh. +bFGF+AChR^+^ sorting	Myoblast-like Pax3^+^, Pax7^+^	No	-	-	[43]
hiPSC	Piggyback transposon inducible MyoD1	Myoblast-like MyoD1^+^	- Immuno-deficient diabetic - Intramuscular - Cardiotoxin	Low numbers of positive fibers	NA	[32]
miPSC dKO	Inducible Pax3 expression on EB +PDGFαR^+^FLK1^−^ selection +μUTR gene correction	Myoblast-like Pax3^+^	- dKO dystrophin—utrophin mice - Immunosuppr ession - Intramuscular (1) - Intra-arterial (1)	- 20% fibers positive (1). - Muscle colonization (2) - Functional recovery (1,2)	Yes	[40]
hiPSC BMD ^&^, SMA, ALS	Free floating spherical culture +FGF2, EGF	Myoblast-like	-	-	-	[42]

* NA = not assessed; ** Duchenne’s Muscular Dystrophy; ^&^ Becker’s Muscular Dystrophy; Ref.: Reference.

## 4. Disease Modeling

The different approaches published so far to make myogenic progenitors from hiPSCs are good models of myogenesis *in vitro*, as the produced cells recapitulate the expression of markers observed *in vivo*. They are able to fuse to produce premature myofibers in the animal *in vitro* and in most cases they have been tested in animal models for engrafting and fusion with host fiber. Several reports describe the establishment of myogenic cell lines produced from iPSCs from patients with different types of muscular dystrophy. Human iPSC-MPs have been established using MyoD1 overexpression by a PiggyBac vector on hiPSCs: Miyoshi Myopathy, a distal myopathy caused by mutations in DYSFERLIN, patients’ fibroblasts [42], and carnitine palmitoyltransferase II deficiency, is an inherited disorder that leads to rhabdomyolysis [47]. Duchenne muscular dystrophy, the most common type of MD, is due to a mutation in the dystrophin gene and has been modeled by adenoviral expression of MyoD1 [41] and by inducible lentiviral Pax3 overexpression [40]. The group of Hosoyama have also described the derivation of myogenic derivatives using their sphere-base culture system from hiPSCs from Becker’s muscular dystrophy, spinal muscular atrophy, and amyotrophic lateral atrophy [42]. The created cell lines make great tools for drug screening and further research into the molecular mechanisms of the different myopathies, and can be obtained in large quantities with minimal patient invasion.

## 5. Future Challenges for Clinical Application

Myogenic progenitors made from iPSCs seem to be a promising candidate for stem cell therapy to regenerate skeletal muscle since they can be produced in large quantities and present more embryonic-like features, so are probably more motile and proliferative compared to adult myoblasts. However, even though results in animal models show an improvement from previous work with isolated myoblasts, in terms of fiber contribution and functional recovery [39,41], a clinically relevant transplantation protocol still needs to be designed.

### 5.1. In Vivo Survival, Engraftment and Migration

One of the major caveats of myoblast therapy was the massive death after transplantation. The inflammatory and immunological response to allogenic transplants probably played a role in the survival of the cells and also engraftment, migration, and differentiation [48]. However, myoblast death is seen before the onset of the immunological response and in the presence of immunosuppressors or for autologous transplantation, where there should be no immune response [21,23]. Also, anoikis and the toxic environment from the high oxidant stress that characterizes dystrophic muscles may play a role in the survival of cells. These challenges to survival will be encountered by hiPSCs-MP in the same ways as purified adult myoblasts. Regarding engraftment, all the published work on hiPSCs-MP in animal models shows *in vivo* engraftment and fusion with host cells, but greater extent is needed for a clinically relevant cell therapy protocol. Limited migration from the injection site, in part due to high mortality, but also to intrinsic capacity, is another major limitation that iPSC-derived cells must overcome to outperform myoblast therapy. Some authors describe iPSC-MP as resembling embryonic more than adult myoblasts [31]. The use of two markers expressed during embryogenesis by hypaxial migratory myogenic precursors, C-MET and CXCR4, has been proposed to isolate the most migratory fraction of hiPSC-MD [49]. Also, beta 1 integrin, expressed in satellite cells, is essential for engraftment [11] and can be another migratory phenotype selection marker.

### 5.2. Fibrosis

Another major limitation to regeneration is dense fibrotic tissue. TGF-β1 induces collagen I deposition from myogenic cells with subsequent fibrotic tissue formation. Fibrosis limits myoblast engraftment as well as motility and this prevents axons from arriving to myofibers. Unfortunately, there are no drugs on the market that can overcome fibrosis in MD patients. However, there is a report that bone marrow-derived stromal cell transplantation in the muscle of an ischemia model reduced fibrosis due to paracrine effects [50]. This inhibitory effect should be studied in hiPSCs-MP if they are to be a candidate for use in a clinical setting. 

### 5.3. Creating the Perfect Niche

Tissue engineering can also be of great help for the survival of transplanted myogenic progenitors in the hostile environment of a damaged tissue. Creating a three-dimensional niche for the transplanted myogenic progenitors that resembles satellite cells’ natural niche *in vivo* by using biomaterials (alginate, collagen, and hyaluran) will conserve the engrafted cells’ homeostasis and allow asymmetric division and myogenic commitment [51]. The cells to be transplanted would be seeded in the 3D scaffold and a graft generated *in vitro*. To complete the niche, extracellular matrix components and signaling molecules to stimulate proliferation, migration, and angiogenesis should be included. Muscle flaps made with decellularized devices from large mammals and synthetic scaffolds complemented with an *in vitro*-produced extracellular matrix from cell cultures derived from the host provide suitable tools for translation to the clinic [52]. From the complex set of requirements for skeletal muscle tissue engineered implants to function and integrate *in vivo*, some issues have already been addressed, such as restoration of the muscular-tendon junction or vascularization, while others like reinnervation still need further work [49].

### 5.4. Genetic Correction vs. Immunocompatible Transplantation

When addressing genetic origin myopathies, the transplanted cells should contain the correct version of the gene. This can be achieved in two ways: by genetic correction of patient-derived cells or by allogenic transplantation of immunocompatible donor cells. One of the major features of iPSCs is the possibility of generating patient-derived tissues with minor invasion. Several groups have performed gene correction on patient iPSCs. iPSC-derived mesangioblasts, from a Limb-Girdle MD patient, in which the wild-type alpha-sarcoglycan gene had been restored by lentiviral delivery, engrafted, and fused with host fibers when transplanted in nude mice [46]. Lamin A/C (LMNA) has also been corrected in laminopathy patient-derived iPSCs using a helper-dependent adenoviral vector, which is safer than other viral vector approaches [53]. Duchenne MD iPSCs have also been corrected with μ-utrophin using a sleeping beauty transposon system [39]. In any case, gene therapy is still under development and a totally safe way of gene correction has still not been demonstrated. 

Another approach is to transplant cells created from a healthy donor that are matched for the main antigens in the host immunological rejection, the HLA antigens. An HLA-typed bank of iPSCs could be created to provide a source of compatible donor cells for the individual patients. A relatively small number of donors can provide an acceptable match to a high percentage of the population [54]. This approach would also be more feasible as a therapeutic approach than the expensive and time-consuming generation of personalized iPSC-MP.

It is necessary to take into account that in the case of genetic diseases that lack the native protein, its expression from the grafted tissue will most likely induce a considerable immune response that needs to be carefully addressed.

### 5.5. Delivery Route

Moreover, the desirable myogenic progenitor should be able to cross the blood barrier to allow for systemic delivery. Treatment of local damage could be done by local intramuscular injections or bio-engineered grafts, but for a cell therapy for MD, SMA, and ALS, in which all muscles in the body are affected, a systemic delivery is necessary. Very few reports show successful engraftment after intra-arterial delivery [38,39,46]. The adequate dosage and regime of injections still needs further study.

### 5.6. Safety

For all the reported work in humans and animals models using muscle stem cells, neither adverse side effect has been described, nor colonization in other organs when systemically delivered [39]. Also, for iPSC-MP no teratoma formation has been detected [37,39]. However, the double reprogramming process—first to pluripotency and then to myogenic lineage—bring along the risk of chromosomal abnormalities and genetic instability [55]. Darabi *et al.* described how, from several clones tested for *in vivo* engraftment and fiber contribution, those that performed better were the ones with a normal karyotype [38]. In this sense, chromosomal, genetic, and epigenetic studies must be performed on the cells to be transplanted before taking them to the clinic application. Also, reprogramming and differentiation methods should not include exogenous DNA but use, for example, mRNA transfection; the use of the oncogene c-Myc should be avoided when reprogramming for clinical applications. Genes involved in epigenetic remodeling [56] and cell cycle regulation [57] have been proposed as alternatives to c-Myc in reprogramming. In this regard, variants of c-Myc with no oncogenic potential such as L-Myc or the W136E c-Myc mutant are also able to induce reprogramming to pluripotency with less tumorigenic potential [58].

### 5.7. Clinical Grade Protocols

Whatever the method of choice is for generating the myogenic progenitors, a clinical grade protocol must be designed for the cells to be used in patients. The generation process should not include any viral vector or exogenous DNA, should be free of animal products, and should use as far as possible defined media to increase reproducibility and comply with good manufacturing procedures. Such a protocol has not yet been described for either iPSC generation or the derivation of MP.

## 6. Conclusions

The use of hiPSCs as a source of myogenic progenitors for cell therapy for the treatment of muscle degenerative diseases overcomes several of the limitations encountered in adult myoblast therapy: (i) easy non-invasive source of donor cells; (ii) unlimited proliferative capacity *in vitro*, and (iii) better performance when tested in mouse models *in vivo*—possibly because of more embryonic-like features. In recent years, several protocols of derivation of myogenic progenitors from iPSCs have been described reaching very satisfactory efficiency in a short time. The use of transcription factors (Pax7, MyoD1) overexpression or GSK3β inhibitors has contributed greatly in this direction. However, a clinical grade protocol still needs to be described, including the definition of safety and genetic stability requirements for clinical applications. Also, isolation of the MP presenting the most promising features for successful regeneration *in vivo* could improve the performance of the cell therapy, such as selecting cells that are more migratory and proliferative or with the possibility of systemic delivery. Other limitations relating to the host—for example, the inflammatory and immune response and the appearance of fibrotic tissue—present a major hurdle to a cell therapy approach. More research with selective inhibitors or modulators of these processes is needed, and the use of bioengineering to create a 3D protective niche for the transplanted cells would contribute to the long-term success of a muscle stem cell therapy strategy. 
